# Exposure to gender-based violence and depressive symptoms among transgender women in Cambodia: findings from the National Integrated Biological and Behavioral Survey 2016

**DOI:** 10.1186/s13033-018-0206-2

**Published:** 2018-05-29

**Authors:** Siyan Yi, Sovannary Tuot, Srean Chhim, Pheak Chhoun, Phalkun Mun, Gitau Mburu

**Affiliations:** 1 KHANA Center for Population Health Research, No. 33, Street 71, Phnom Penh, Cambodia; 2 Center for Global Health Research, Touro University California, Vallejo, USA; 3 FHI 360, Phnom Penh, Cambodia; 4 National Center for HIV/AIDS, Dermatology and STD, Phnom Penh, Cambodia; 5 Division of Health Research, Lancaster University, Lancaster, UK

**Keywords:** Depression, Gender-based violence, Transgender women, Mental health, National survey, Cambodia

## Abstract

**Background:**

Transgender women are at significant risk of HIV, and they face intersecting barriers to health, social, and legal services. However, data regarding the unique needs and experiences of transgender women are globally scant. This study examined the relationship between gender-based violence and depressive symptoms among transgender women in Cambodia.

**Methods:**

This cross-sectional study included 1375 sexually active transgender women recruited by using the respondent-driven sampling method in the capital city of Phnom Penh and 12 provinces between December 2015 and February 2016. Depressive symptoms were assessed using the Center for Epidemiologic Studies Depression scale (CES-D). Multivariate regression analysis was conducted to explore factors independently associated with depressive symptoms.

**Results:**

Of total, 45.0% of the participants had depressive symptoms, and 21.8% had severe depressive symptoms. After controlling for potential confounders, transgender women with depressive symptoms remained significantly more likely to report several negative experiences of gender-based violence such as a feeling that co-workers or classmates were not supportive regarding their transgender identity (AOR = 2.00, 95% CI = 1.22–3.28), having difficulties in getting a job (AOR = 1.67, 95% CI = 1.29–2.16), having been denied or thrown out of housing (AOR = 1.53, 95% CI = 1.02–2.26), having difficulties in getting health services (AOR = 2.40, 95% CI = 1.50–3.82), having been physically abused (AOR = 1.54, 95% CI = 1.15–2.08), and having been fearful of being arrested by police or authorities (AOR = 2.18, 95% CI = 1.64–2.91) because of their transgender identity. Regarding their childhood experiences, transgender women with depressive symptoms remained significantly more likely to report that someone had tried to touch them or make them touch in a sexual way when they were growing up (AOR = 2.08, 95% CI = 1.61–2.68).

**Conclusions:**

Transgender women in Cambodia experience high levels of gender-based violence and depressive symptoms. To address these concerns, a combination of service and policy interventions are required. These may include training and sensitization of trained and lay health providers in screening for depressive symptoms and integration of mental health services into facility- and community-based HIV services with enforcement of policies and laws that protect the rights of transgender women against gender-based violence.

## Background

Poor mental health is a leading cause of human morbidity and mortality [1, 2]. Worldwide, transgender women experience a variety of mental health problems, due to a range of intersecting reasons. Previous research in different settings suggests that transgender women are exposed to high levels of stigma and discrimination due to their transgender identity [3, 4]. In addition, legal marginalization of transgender women provides contexts that aid arbitrary arrests, stigma, and abuse of transgender women in different countries [5–7].

More recently, researchers have started investigating the confluence of different forms of marginalization on the health and wellbeing of transgender women [8]. In Colombia, a recent study showed how the intersection of conflict, violence, homonegativity, and ‘social cleansing’ increase the vulnerability of displaced transgender women to HIV [9]. In India, the intersection of transgender victimization and mental health was shown to increase substance abuse and HIV vulnerability among transgender women [10].

Globally, HIV prevalence among transgender women is much higher than that among the general population, currently estimated at 19.1% [11]. In the context of HIV, consideration of mental health status of vulnerable populations is important, given that depression and other mental health problems are known to increase uptake of sexual risk taking [10–13] and substance abuse [14], which can in turn increase HIV transmission [15–17]. The intersecting influences of substance abuse, sexual risk taking, and depression often occur alongside physical, sexual, and gender-based violence in a cycle that further increases risk to HIV infection [10, 14, 18]. Furthermore, according to a systematic review and meta-analysis of studies in low-, middle- and high-income countries, untreated depression reduces optimal adherence to ART among people living with HIV [19].

According to our recent National Integrated Biological and Behavioral Survey, HIV prevalence among transgender women in Cambodia was 5.9% in 2016 [20], and they are a priority population for HIV and public health interventions [21]. Despite the prioritization of transgender women in the Cambodian HIV response, there is a dearth of strategic information and epidemiological data, which can support decision-making. Until recently, transgender women in Cambodia had been included in statistics relating to men who have sex with men, making it difficult to identify their unique health needs or develop tailored services [21, 22]. Although studies of transgender women in Cambodia have started to emerge [20, 22–24], they understandably tend to focus on proximal epidemiological indicators and drivers of HIV, relatively ignoring distal determinants of HIV transmission such as gender-based violence and mental health problems. In response to this gap, this study was conducted to examine the relationship between gender-based violence and depressive symptoms among transgender women in Cambodia.

## Methods

### Study sites and participants

This cross-sectional study was conducted between December 2015 and February 2016 in the capital city of Phnom Penh and 12 provinces of Cambodia. The Respondent Driven Sampling (RDS) method was used to recruit participants. Among the 13 study sites, participants were recruited in 20 specific locations (six locations in Phnom Penh and 14 locations in the provinces). The number of the selected locations was determined based on the proportion of the required sample size and the estimated population size of transgender women in each study site. People would be included in the study if they: (1) were aged 18 years or older, (2) were biologically male at birth and self-identified as a woman, (3) reported having sex with at least one man in the past 12 months, (5) could speak Khmer, and (6) were able and willing to provide a written informed consent to participate in the study.

Four initial seeds were identified at each location by outreach workers from KHANA’s implementing partners based on age (two seeds aged 18–24 and the other two seeds aged 25 or older). These seeds had to meet the eligibility criteria and have an established social network comprising about 10 or more other transgender women in their given location. Eligibility to participate as a seed was determined by the leader of data collection team using a paper-based eligibility form. Each seed was given three coupons and asked to refer three additional transgender women. Each seed would receive US$2 for a successful referral and was expected to extend to 3–6 “recruitment waves” in each location. If the initial seeds did not recruit participants or if the enrollment was halted because all recruitment chains had “dried up” (i.e. stopped recruiting), additional seeds would be selected based on the above criteria. In total, 80 seeds were initially selected.

### Data collection training and procedures

Prior to the data collection, 3 days of training were provided to all interviewers and field supervisors on data collection methods and tool pretesting to ensure quality of the data. The training included interview techniques, confidentiality and privacy, and provided opportunities for the study teams to rehearse questionnaire administration and other study procedures. During data collection, review sessions with interviewers were conducted regularly to review progress and communicate any problems.

Two data collection teams were formed with eight personnel each that included one field supervisor, five interviewers, one lab technician, and one counselor from the Provincial AIDS and STD Program. The field supervisor conducted eligibility screening of the participants. Each consenting participant was assigned a unique personal identification number, which was used to link all data collected from each participant. The unique personal identification number was not linked with any personal data to protect confidentiality. The counselor then explained the objectives of the study in details, including the process of HIV testing and potential risks and benefits of participation. After obtaining an informed consent, an interviewer administered the questionnaire in a private room using an Android tablet. Each interview took between 30 and 40 min. Each participant received US$4 in cash to compensate for their time and transport, and were given a package of three condoms.

### Questionnaire development

The questionnaire was initially developed in English and then translated into Khmer, the national language of Cambodia. Another translator then back-translated it into English to ensure that the “content and spirit” of every original item was maintained. Clear instructions and explanations were included to avoid any confusion during the interviews. Consultative meetings were held with representatives of transgender women and key stakeholders working on HIV key populations in Cambodia. Prior to data collection, the questionnaire was pretested to ensure that the wording and contents of the questionnaire were culturally suitable, acceptable, and clearly understood by the study participants before it was finalized. The pilot study was conducted with 20 transgender women in Phnom Penh to assess the contents, format, length, language, and appropriateness of the questionnaire. Necessary modifications were made based upon feedbacks from the pilot study and from the consultative meetings.

Socio-demographic characteristics included study sites (urban, rural), age, perceived gender identity, marital status, main occupation, average income in the past 6 months, duration living in the current city, completed years of formal education, and perceived family economic status. We also collected information regarding gender expression and utilization of gender affirming hormones and surgeries. Most of the items were adapted from the most recent Cambodia Demographic and Health Survey [25] and our previous community-based surveys among HIV key populations in Cambodia [24, 26, 27].

Experiences of gender-based violence were measured using items adapted from previous studies [12, 28]. Participants reported different forms of discrimination and violence they had experienced in the past 12 months. These included whether the participants felt that their co-workers or classmates are supportive regarding their transgender identity and had experienced problems such as difficulties in getting a job, losing a job, having been denied or thrown out of a housing, and difficulties in getting HIV or other health services and thought it was because of their transgender identity. Participants were also asked about their experiences in different forms of violence such as having been physically or sexually abused, been arrested, dropped out of school, and been fearful of being arrested by police or authorities (Cronbach’s alpha = 0.74).

Five questions were adapted from the brief screening version of the Childhood Trauma Questionnaire to measure adverse childhood experiences [29]. The five questions asked about the experiences of physical abuse, emotional abuse, sexual abuse, physical neglect, and emotional neglect during the time when they were growing up. The response options for each question ranged from (1) ‘never’ to (5) ‘very often’. Participants who responded ‘never’ and ‘rarely’ were grouped together as those without adverse childhood experiences. Participants who answered ‘sometimes’, ‘often’, and ‘very often’ were grouped together as those with adverse childhood experiences.

We used the Center for Epidemiologic Studies Depression Scale (CES-D) to measure depressive symptoms [30]. This scale consists of 20 questions addressing six symptoms of depression including depressed mood, guilt or worthlessness, helplessness or hopelessness, psychomotor retardation, loss of appetite, and sleep disturbance experienced during the preceding week. Each question is scored on a scale of 0–3 according to the frequency of the symptoms, and the total CES-D score ranges from 0 to 60. To calculate the total score, four items (I felt I was just as good as other people, I felt hopeful about the future, I was happy, I enjoyed life) were reverse coded. Cronbach’s alpha among participants in this study was 0.88. The criterion validity of the CES-D scale has been well established in Western [30] and Asian [31] populations. Depressive symptoms are defined as present when a subject had a CES-D score of ≥ 16. A cutoff value of ≥ 23 was also used to define severe depressive state [32].

### Data analyses

We used EpiData version 3 (Odense, Denmark) for double data entry, and SPSS version 22 (IBM Corporation, New York, USA) for all statistical analyses. In bivariate analyses, χ^2^ test (or Fisher’s exact test when the sample sizes were smaller than five in one cell) was used for categorical variables and Student’s *t* test for continuous variables to compare socio-demographic characteristics, experiences of gender-based violence, and adverse childhood experiences among transgender women with depressive symptoms, defined by a CES-D score of ≥ 16, to those without depressive symptoms.

A multivariate logistic regression model was constructed to control for potential confounding factors. All variables significantly associated with depressive symptoms in the bivariate analyses at a level of *p*-value < 0.05 were first simultaneously included in the model. Then variables with a *p*-value > 0.05 were removed, and the models were refitted. We repeated the steps until all *p*-values of the remaining variables were < 0.05 in the final model. Adjusted odds ratio (AOR) were obtained and presented with confidence interval (CI) and *p*-values.

### Ethical considerations

The study protocol was approved by the National Ethics Committee for Health Research (NECHR) of the Ministry of Health, Cambodia (No. 420 NECHR) and FHI 360′s Protection of Human Subjects Committee (PHSC No. 713897). A written informed consent was obtained from each participant after details about the study objectives, risk, and benefits had been explained to them. We also informed the participants that they could withdraw from the study at any time. Privacy of participants was protected by conducting the interviews in a private room. We also ensured confidentiality by assigning a personal identity number (PIN) to each participant and removing all personal identifiers.

## Results

### Socio-demographic characteristics

This study included 1375 transgender women with a mean age of 25.8 (SD = 7.1). The proportion of participants with depressive symptoms and severe depressive symptoms were 45.0 and 21.8%, respectively. The majority of participants were not married or living with a partner (78.1%) and had an average monthly income of US$186 (SD = 231) and a mean completed years of formal education of 9.0 (SD = 3.4). More than one-third of them (35.1%) worked as a hairdresser or beautician. Less than half (42.3%) self-identified as female; 45.0% had used gender affirming hormone; and 9.3% had had gender-affirming surgeries (Table 1).

**Table 1:** Comparisons of socio-demographic characteristics, gender identity, and gender-related experiences of transgender women with and without depressive symptoms

Characteristics	Total (n = 1375)	Depressive symptoms^a^		
		No (*n *= 756)	Yes (*n *= 619)	p-value^†^
Study site				0.002
Urban	1146 (83.3)	651 (56.8)	495 (43.2)	
Rural	229 (16.7)	105 (45.9)	124 (54.1)	
Age group				0.04
18–24	729 (53.0)	423 (58.0)	306 (42.0)	
25–34	503 (36.6)	264 (52.5)	239 (47.5)	
≥ 35	143 (10.4)	69 (48.3)	74 (51.7)	
Marital status				0.001
Married	7 (0.5)	4 (57.1)	3 (42.9)	
Widowed/divorced/separated	18 (1.3)	7 (38.9)	11 (61.1)	
Not married/not living with a partner	1074 (78.1)	622 (57.9)	452 (42.1)	
Not married/living with a partner	260 (18.9)	114 (43.8)	146 (56.2)	
Months living in current city	16.4 ± 11.3	16.5 ± 11.2	16.4 ± 11.3	0.91
Monthly income (USD)	186 ± 231	189 ± 228	182 ± 235	0.58
Years of education completed	9.0 ± 3.4	9.4 ± 3.3	8.5 ± 3.4	< 0.001
Current job (main source of income)				< 0.001
Unemployed	64 (4.7)	33 (51.6)	31 (48.1)	
Hair dresser/beautician	482 (35.1)	242 (50.2)	240 (49.8)	
Government officer	16 (1.2)	11 (68.8)	3 (31.3)	
Laborer	222 (16.1)	126 (56.8)	96 (43.2)	
Seller	149 (10.8)	79 (53.0)	70 (47.0)	
Entertainment worker	140 (10.2)	82 (58.6)	58 (41.4)	
Sex worker	27 (2.0)	9 (33.3)	18 (66.7)	
Student	108 (9.7)	84 (77.8)	24 (22.2)	
NGO staff	34 (2.5)	19 (55.9)	15 (44.1)	
Private company staff	34 (2.5)	21 (61.8)	13 (38.2)	
Farmer/fisherman	19 (1.4)	9 (47.4)	10 (52.6)	
Artist	36 (2.6)	21 (58.3)	15 (41.7)	
Other	44 (3.2)	20 (45.5)	24 (54.5)	
Gender self-identity				< 0.001
Female	581 (42.3)	284 (48.9)	297 (51.1)	
Third gender	794 (57.7)	472 (59.4)	322 (40.6)	
Frequency of expressing as a woman				0.36
All the time	661 (48.1)	355 (53.7)	306 (46.3)	
Not all the time	714 (51.9)	401 (56.2)	313 (43.8)	
Ever used gender-affirming hormones				< 0.001
No	756 (55.0)	451 (59.7)	305 (40.3)	
Yes	619 (45.0)	305 (49.3)	314 (50.7)	
Ever had gender affirming surgeries				0.32
No	1247 (90.7)	691 (55.4)	556 (44.6)	
Yes	128 (9.3)	65 (50.8)	63 (49.2)	

Values are numbers of subjects (%) for categorical variables and mean ± standard deviation (SD) for continuous variables

^†^ Chi square test was used for categorical variables; independent Student’s t-test was used for continuous variables

^a^ Defined by a Center for Epidemiology Studies Depression Scale (CES-D) score of ≥ 16

Table 1 also shows that the proportion of transgender women with depressive symptoms was significantly higher among participants who were living in rural areas, in older age groups, widowed/divorced/separated, with lower a level of formal education, sex workers, farmers, unemployed, self-identified as female, and using gender-affirming hormones.

### Experiences of gender-based violence

As shown in Table 2, a fairly large proportion of transgender women in this study had experienced different forms of gender-based violence because of their transgender identity or expression. The experiences included a feeling that their co-workers or classmates were not supportive regarding transgender identity (10.1%), difficulties in getting a job (41.9%), job loss (24.1%), having been denied or thrown out of a housing (18.8%), physical abuse (23.6%), sexual abuse or assault (39.3%), dropping out of school (24.1%), and being fearful of being arrested by police or authorities (24.8%) because of their transgender identity.

**Table 2:** Comparisons of experiences of gender-based violence among transgender women with and without depressive symptoms

Experiences of ender-based violence	Total (n = 1375)	Depressive symptoms^a^		
		No (*n* = 756)	Yes (*n* = 619)	p-value^†^
Co-workers or classmates are supportive regarding transgender identity				< 0.001
Very much supportive	215 (15.7)	121 (56.3)	94 (43.7)	
Supportive	1013 (74.1)	580 (57.3)	433 (42.7)	
Not supportive	129 (9.4)	50 (38.8)	79 (61.2)	
Not at all supportive	10 (0.7)	3 (30.0)	7 (70.0)	
Difficulties in getting a job because of transgender identity				< 0.001
No	794 (51.8)	528 (66.5)	266 (33.5)	
Yes	573 (41.9)	226 (39.4)	347 (60.6)	
Lost a job because of your transgender identity				< 0.001
No	1037 (75.9)	645 (62.2)	392 (37.8)	
Yes	330 (24.1)	109 (33.0)	221 (67.0)	
Denied/thrown out of a housing because of your transgender identity				< 0.001
No	1121 (81.0)	673 (60.0)	448 (40.0)	
Yes	246 (18.0)	81 (32.9)	165 (67.1)	
Difficulties in getting HIV services because of your transgender identity				< 0.001
No	1252 (91.6)	724 (57.8)	528 (42.2)	
Yes	115 (8.4)	30 (26.1)	85 (73.9)	
Difficulties in getting health services because of your transgender identity				< 0.001
No	1243 (90.9)	725 (58.3)	518 (41.7)	
Yes	124 (9.1)	29 (23.4)	95 (76.6)	
Physically abused because of your transgender identity				< 0.001
No	1045 (76.4)	638 (61.1)	407 (38.9)	
Yes	322 (23.6)	116 (36.0)	206 (64.0)	
Sexually abused or assaulted because of your transgender identity				< 0.001
No	830 (60.7)	515 (62.0)	315 (38.0)	
Yes	537 (39.3)	239 (44.5)	298 (55.5)	
Arrested because of your transgender identity				< 0.001
No	1224 (89.5)	711 (58.1)	513 (41.9)	
Yes	143 (10.5)	43 (30.1)	100 (69.9)	
Dropped school because of your transgender identity				< 0.001
No	1037 (75.9)	622 (60.0)	415 (40.0)	
Yes	330 (24.1)	132 (40.0)	198 (60.0)	
Fearful of being arrested by police/authorities because of transgender identity				< 0.001
No	1028 (75.2)	635 (61.8)	393 (38.2)	
Yes	339 (24.8)	119 (35.1)	220 (64.9)	

Values are numbers of subjects (%) for categorical variables

^†^ Chi square test was used for categorical variables or Fisher’s exact test was used as appropriate

^a^ Defined by a Center for Epidemiology Studies Depression Scale (CES-D) score of ≥ 16

Table 2 shows that the proportion of transgender women with depressive symptoms was significantly higher among participants who perceived that their co-workers or classmates were not supportive regarding their transgender identity and those who reported having difficulties in getting a job, lost a job, been denied or thrown out of a housing, difficulties in getting HIV and other health care services, been physically and sexually abused or assaulted, dropped out of school, and been fearful of being arrested by police or authorities because of their transgender identity.

### Adverse childhood experiences

Table 3 shows that adverse childhood experiences were common among transgender women in this study. They reported that they had been hit, slapped, or kicked by a parent or guardian (58.7%); that people in their family had said hurtful or insulting things to them (64.4%); and that someone had tried to touch them or make them touch in a sexual way, make them do or watch sexual things, or actually did something sexual with them (32.5%) when they were growing up. The proportion of transgender women with depressive symptoms was significantly higher among those who reported physical, emotional, and sexual abuse and lower among those who reported positive childhood experiences such as having someone to take care of them and take them to medical care when they got sick and someone who helped them feel that they were loved and important compared to those who did not (Table 3).

**Table 3:** Comparisons of adverse childhood experiences among university students with and without depressive symptoms

Adverse childhood experiences	Total (*n *= 1375)	Depressive symptoms^a^		
		No (*n *= 756)	Yes (*n *= 619)	p-value^†^
Had been hit, slapped, or kicked by a parent or guardian				< 0.001
No	568 (41.3)	347 (61.1)	221 (38.9)	
Yes	807 (58.7)	409 (50.7)	398 (49.3)	
People in my family had said hurtful or insulting things to me				< 0.001
No	489 (35.6)	313 (64.0)	176 (36.0)	
Yes	886 (64.4)	443 (50.0)	443 (50.0)	
Someone had tried to touch me or make me touch them in a sexual way				< 0.001
No	928 (67.5)	580 (62.5)	348 (37.5)	
Yes	447 (32.5)	176 (39.4)	271 (60.6)	
There had been someone to take care of me when I got sick				0.003
No	43 (3.1)	14 (32.6)	29 (67.4)	
Yes	1332 (96.9)	742 (55.7)	590 (44.3)	
There had been someone who helped me feel that I was loved and important				0.007
No	104 (7.6)	44 (42.3)	60 (57.7)	
Yes	1271 (92.4)	712 (56.0)	559 (44.0)	

Values are numbers of subjects (%)

^†^ Chi square test was used

^a^ Defined by a Center for Epidemiology Studies Depression Scale (CES-D) score of ≥ 16

### Factors associated depressive symptoms

Results of multivariate logistic regression analyses are shown in Table 4. After adjustment for other covariates in the model, transgender women with depressive symptoms remained significantly more likely to live in a rural area (AOR = 1.55, 1.13–2.12) and less likely to be an entertainment worker (AOR = 0.52, 95% CI = 0.27–0.99) and student (AOR = 0.34, 95% CI = 0.17–0.70) compared to their respective comparison group. Transgender women with depressive symptoms also remained significantly more likely to report several negative experiences of gender-based violence such as a feeling that co-workers or classmates were not supportive regarding their transgender identity (AOR = 2.00, 95% CI = 1.22–3.28), having difficulties in getting a job (AOR = 1.67, 95% CI = 1.29–2.16), having been denied or thrown out of a housing (AOR = 1.53, 95% CI = 1.02–2.26), having difficulties in getting health services (AOR = 2.40, 95% CI = 1.50–3.82), having been physically abused (AOR = 1.54, 95% CI = 1.15–2.08), and having been fearful of being arrested by police or authorities (AOR = 2.18, 95% CI = 1.64–2.91) because of their transgender identity. Regarding their childhood experiences, transgender women with depressive symptoms remained significantly more likely to report that someone had tried to touch them or make them touch in a sexual way when they were growing up (AOR = 2.08, 95% CI = 1.61–2.68).

**Table 4:** Factors associated with depressive symptoms among transgender women (*n *= 1375)

Variables in the final model^a^	Depressive symptoms^b^	
	AOR (95% CI)	p-value
Study site		
Urban	Reference	
Rural	1.55 (1.13–2.12)	0.006
Main occupation		
Unemployed	Reference	
Entertainment worker	0.52 (0.27–0.99)	0.04
Student	0.34 (0.17–0.70)	0.003
Co-workers or classmates are supportive regarding your transgender identity		
Very much supportive	Reference	
Supportive	1.08 (0.78–1.51)	0.63
Not supportive	2.00 (1.22–3.28)	0.006
Not at all supportive	1.78 (0.39–8.20)	0.46
Difficulties in getting a job because of your transgender identity		
No	Reference	
Yes	1.67 (1.29–2.16)	< 0.001
Denied or thrown out of a housing because of your transgender identity		
No	Reference	
Yes	1.53 (1.02–2.26)	0.04
Difficulties in getting health services because of your transgender identity		
No	Reference	
Yes	2.40 (1.50–3.82)	< 0.001
Physically abused because of your transgender identity		
No	Reference	
Yes	1.54 (1.15–2.08)	0.04
Fearful of being arrested by police or authorities because of your transgender identity		
No	Reference	
Yes	2.18 (1.64–2.91)	< 0.001
Someone had tried to touch me or make me touch them in a sexual way during childhood		
No	Reference	
Yes	2.08 (1.61–2.68)	< 0.001

*AOR* adjusted odds ratio, *CI* confidence interval

^a^Variables in the table were the ones that remained statistically significant in the final multivariate logistic regression model after several steps of model fitting

^b^Defined by a Center for Epidemiology Studies Depression Scale (CES-D) score of ≥ 16

## Discussion

This study explored factors associated with depressive symptoms among transgender women in Cambodia. The findings are significant in that they show a high prevalence of experiences of gender-based violence and depressive symptoms among this vulnerable population. The prevalence of depressive symptoms and severe depressive symptoms were 45.0 and 21.8%, respectively. These findings echo those from two studies from the United States and China, showing high levels (35–49.8%) of clinical depression symptoms among transgender women [33–36].

Apart from providing important information regarding prevalence of depressive symptoms, this study identified a number of factors, which were independently associated with depressive symptoms including exposure to gender-based violence. First, our findings suggest that residential areas could have an impact of mental health. In our study, transgender women with depressive symptoms were about two times more likely to live in a rural area, probably because of less openness of transgender expression among these women. While other studies have shown an association between geographic location and depression in transgender individuals [37], it is possible that people in rural communities may be less tolerant of gender diversity, causing higher levels of distress to rural transgender women.

Second, it is not surprising that transgender women with depressive symptoms were significantly more likely to report real or perceived experiences of gender-based discrimination and stigmatizing attitudes. In our study, transgender women reported having been discriminated and excluded from accessing essential services and opportunities such as employment and housing services. Transgender women with depressive symptoms were also more likely to have experienced difficulties in accessing health services. In addition to being denied these services and opportunities, which is also reported among transgender women in other countries [5], our study suggests that these discriminatory experiences significantly increase the odds of having depressive symptoms by 1.5 o 2.4 fold. Similar associations between stigma and discrimination victimization on one hand and depression on the other have also been reported in the United States and Canada [3, 8, 36, 38].

Third, among those who are in employment and schools, lack of support from co-workers or classmates regarding their transgender identity doubled the odds of experiencing depressive symptoms. Transgender women with depressive symptoms were more likely to live in fear of arbitrary arrests by police or authorities because of their transgender identity. This is consistent with studies showing that the lack of social support and community belonging increases depressive symptomatology and perceived stress among transgender women in other settings [3], possibly due to minority stress [39].

Fourth, linked to the above point regarding social support, the prevalence of depressive symptoms were lower among transgender women who were entertainment workers or students compared to those in other occupation categories. This might be related to social support that entertainment workers or students might have access such as peer-to-peer social support among themselves, which can be protective of stigma and minority stress, as discussed later.

Finally, our findings also suggest that adverse childhood experiences can have an impact on future mental health problems. In our study, transgender women with depressive symptoms were twice as likely to report that someone had tried to touch them or make them touch in a sexual way when they were growing up. Similar findings of associations between psychological distress and history of traumatic childhood experiences have been reported among other HIV key populations in Cambodia [40–42], and point to the known importance of supportive peers and family in mental wellbeing of these key populations in the country as well as in other settings [43].

### Implications for policy and services

These findings have several implications on policy and services for transgender women in Cambodia. First, HIV services need to be strengthened and adapted so as to identify and be responsive to mental health needs of transgender women in the country. This can be achieved by providing training for the existing health workforce and through integration of principles of gender inclusiveness and evidence about the harmful effects of exclusion into university curricula for health professionals. This has been achieved in other resource-limited settings [44, 45]. In Cambodia, fear of stigmatizing attitudes is a documented barrier of access to mental health services [46], and in our study, transgender women with depressive symptoms were more likely to have experienced difficulties in accessing health services. Therefore, training and sensitization on mental health is essential to ensure that trained health providers can competently provide mental health services without stigmatizing attitudes. Because HIV services are already targeting and reaching transgender women, integrating mental health interventions such mental health counseling would be cost effective. Integration of mental health services will also be beneficial as it can facilitate the identification of people who experiencing gender-based violence, which in our study is associated with depressive symptoms, and which is itself a driver of HIV transmission [47]. To counter stigma and minority stress, peer support interventions from other transgender women should be strengthened. Studies suggest that peer-to-peer support can enhance ability to cope with adversity [43] and ameliorate their experiences of mental distress, depression, and stigma [5, 10, 33, 35, 48]. Conversely, absence of social support can aggravate depressive symptoms [3], suggesting that it should be a core part of interventions for marginalized transgender women. More widely, strengthening peer and social support can also improve other distal treatment outcomes [49–51] and would therefore be a good strategic investment.

Second, further expansion of mental health infrastructure will be required. As is the case in most developing countries, mental health service provision is poorly developed in Cambodia [52, 53]; yet it may not be possible to manage all cases of mental distress among transgender women within HIV services. Complicated cases will need to be referred for specialized mental health services. Given the low ratios of mental health providers in Cambodia [52, 53], further expansion of the mental work force will be essential. Evidence from previous studies in Cambodia suggests that a range of other populations have inadequate access to mental health services [46, 52, 53]. Therefore, expansion of services would also benefit the wider general population.

Third, given the poor reach of mental health services to marginalized populations in Cambodia [46, 52], community-based infrastructure (such as lay providers and drop-in centers) should be used as much as possible to deliver services outside of health facilities. Using a community-based approach will increase the opportunities for reaching marginalized populations with essential mental health services. This will require adapting the existing HIV outreach services so as to include mental health screening and referral as an integral as part of existing peer-led outreach services. Hence the training and capacity building will need to focus on both formal health providers as well as community-based peer educators and outreach workers. This approach will enable integrated mental health and HIV outreach services to reach communities in rural areas. In Cambodia, rural areas have inequitable access to mental health services compared to urban areas [46, 52], yet in our study, rural residents had higher levels of depressive symptoms. Besides achieving equity in coverage, the operationalization of peer-led and community-based mental health and HIV services will also be useful in educating communities, families, authorities regarding transgender women. Educating communities on gender diversity is essential in reducing transgender stigma and HIV vulnerability [54].

Finally, at a policy level, improving existing laws and policies could improve mental health of transgender women. Macro level collaboration involving the government, civil society, and other non-governmental organizations to advocate for the health and rights of transgender women can have a significant effect in mitigating stigma. In our study, transgender women with depressive symptoms were more likely to live in fear of arbitrary arrests by police or authorities because of their transgender identity. Specific policy stance that opposes systematic exclusion and denial of employment, housing, schooling, and other social amenities based on transgender identity will need to be strengthened.

### Study limitations

This was a cross sectional study, and therefore causation may not be inferred from our findings. Our sampling strategy focused on regions with highest concentration of transgender women and HIV, leaving out other areas with fewer transgender women and lower burden of HIV. Thus our findings may not be generalizable to all transgender women in Cambodia. Indeed, transgender women in areas with a smaller population may feel more socially isolated and could experience higher levels of gender-based violence and depressive symptoms. It is also possible that recruitment bias was introduced because participants were approached through the peer networks of seed informants, many of whom were connected with community-based non-government organizations. Our study utilized a self-reporting questionnaire to gather sensitive data on gender-based violence and mental health, which may have introduced social desirability bias. Taken together, the fore-going limitations suggest that our study may be under-estimating the prevalence of gender-based violence and mental health problems in this population. Nevertheless, our findings are useful for developing mental health services for transgender women in Cambodia.

## Conclusions

Despite the above-mentioned limitations, our findings are useful for development of mental health services for transgender women in Cambodia. We found that poor mental health among transgender women in Cambodia is positively associated with rural residence, experience of different forms of gender-based violence such as physical abuse and social exclusion as well as adverse childhood experiences. To address these concerns, a combination of service and policy interventions are required. These may include training and sensitization of trained and lay health providers in screening for depressive symptoms and integration of mental health services into facility- and community-based HIV services with enforcement of policies and laws that protect the rights of transgender women against gender-based violence.
